# Risk of Dementia Diagnosis After Injurious Falls in Older Adults

**DOI:** 10.1001/jamanetworkopen.2024.36606

**Published:** 2024-09-30

**Authors:** Alexander J. Ordoobadi, Hiba Dhanani, Samir R. Tulebaev, Ali Salim, Zara Cooper, Molly P. Jarman

**Affiliations:** 1 Department of Surgery, Brigham and Women’s Hospital, Boston, Massachusetts; 2 Center for Surgery and Public Health, Brigham and Women’s Hospital, Boston, Massachusetts; 3 The Gillian Reny Stepping Strong Center for Trauma Innovation, Boston, Massachusetts; 4 Department of Surgery, Baystate Medical Center, Springfield, Massachusetts; 5 Division of Aging, Brigham and Women’s Hospital, Boston, Massachusetts

## Abstract

**Importance:**

Emerging evidence suggests that mild cognitive impairment, which is a precursor to Alzheimer disease and related dementias (ADRD), places older adults at increased risk for falls. However, the risk that an older adult develops dementia after experiencing a fall is unknown.

**Objective:**

To determine the risk of new ADRD diagnosis after a fall in older adults.

**Design, Setting, and Participants:**

This retrospective cohort study examined Medicare Fee-for-Service data from 2014 to 2015, with follow-up data available for at least 1 year after the index encounter. Participants included adults aged 66 years and older who experienced a traumatic injury that resulted in an emergency department (ED) or inpatient encounter and did not have a preexisting diagnosis of dementia. Data analysis was performed from August 2023 to July 2024.

**Exposures:**

Experiencing a fall compared with other mechanisms of injury, defined by *International Classification of Diseases, Ninth Revision (ICD-9)* and *ICD-10* external cause of injury codes.

**Main Outcomes and Measures:**

The hazard of new ADRD diagnosis within 1 year of a fall, assessed by performing a Cox multivariable competing risk model that controlled for potential confounders while accounting for the competing risk of death.

**Results:**

The study included 2 453 655 older adult patients who experienced a traumatic injury; 1 522 656 (62.1%) were female; 124 396 (5.1%) were Black and 2 232 102 (91.0%) were White; and the mean (SD) age was 78.1 (8.1) years. The mechanism of injury was a fall in 1 228 847 incidents (50.1%). ADRD was more frequently diagnosed within 1 year of a fall compared with other injury mechanisms (10.6% [129 910 of 1 228 847] vs 6.1% [74 799 of 1 224 808]; *P* < .001). The unadjusted hazard ratio (HR) of incident dementia diagnosis after a fall was 1.63 (95% CI, 1.61-1.64; *P* < .001). On multivariable Cox competing risk analysis, falling was independently associated with an increased risk of dementia diagnosis among older adults (HR, 1.21 [95% CI, 1.20-1.21]; *P* < .001) after controlling for patient demographics, medical comorbidities, and injury characteristics, while accounting for the competing risk of death. Among the subset of older adults without a recent skilled nursing facility admission, the HR was 1.27 (95% CI, 1.26-1.28; *P* < .001).

**Conclusions and Relevance:**

In this cohort study, new ADRD diagnoses were more common after falls compared with other mechanisms of injury, with 10.6% of older adults being diagnosed with ADRD in the first year after a fall. To improve the early identification of ADRD, this study’s findings suggest support for the implementation of cognitive screening in older adults who experience an injurious fall that results in an ED visit or hospital admission.

## Key Points

**Question:**

What is the risk of developing dementia after an older adult experiences a fall compared with other mechanisms of injury?

**Findings:**

In this cohort study involving 2 453 655 older adults who sustained an injury, 10.6% of patients who experienced a fall were subsequently diagnosed with dementia within 1 year. Compared with other injury mechanisms, falling was associated with a 21% increased risk for future dementia diagnosis after controlling for potential confounders.

**Meaning:**

These results suggest that cognitive screening should be implemented for older adults who have experienced a fall that results in an emergency department visit or hospital admission.

## Teaser

This cohort study investigates the risk of a new diagnosis for Alzheimer disease and related dementias after a fall in older adults.

## Introduction

Falls are the most common cause of injury in older adults, with 27.5% of older adults reporting a fall in the past year.^1^ They are also the most common reason for trauma center admission among patients aged 65 years and older.^2^ Older adults who experience a fall face substantial morbidity, decline in functional status, and loss of independence.^3,4,5^ Falls in older adults are also responsible for $50 billion in health care expenditures each year.^6^ Given the substantial morbidity and costs attributed to falls, both the Centers for Disease Control and Prevention (CDC) and the American College of Surgeons have identified fall prevention as a key public health intervention.^7,8^ As clinicians work to prevent falls, patients with cognitive impairment, including those with Alzheimer disease and related dementias (ADRD, hereafter referred to as dementia), have been identified as a group with increased risk for falls.^9^

There is growing evidence of an association between cognitive impairment and falls. Patients with both dementia and mild cognitive impairment, a precursor to dementia, are at increased risk for experiencing falls.^10,11^ In addition, motor impairments may precede the onset of dementia, and these motor impairments can result in an increased risk of falls in the years leading up to the documented diagnosis of dementia.^12,13^ Several studies have also demonstrated that patients who experience hip fractures, a common injury after falls in older adults, are at increased risk for future dementia diagnosis.^14,15^ Despite this link between cognitive impairment and fall risk, current fall prevention guidelines inconsistently recommend cognitive screening.^16^

Given these interactions between falls and cognitive impairment, it is possible that falls serve as a sentinel event that marks a future risk for dementia. Such a finding could aid in the early diagnosis of dementia, which is a priority of the CDC Healthy Brain Initiative.^17^ We therefore performed a retrospective cohort study examining the risk of incident dementia diagnosis among older adults in the US who experienced a traumatic injury, including both fall and nonfall mechanisms of injury. We hypothesized that experiencing a fall is associated with an increased risk for a future diagnosis of dementia among older adults.

## Methods

### Data Source and Study Population

We performed a retrospective cohort study of Medicare claims data for all older adult beneficiaries who were continuously enrolled in Fee-for-Service Medicare and were diagnosed with a traumatic injury resulting in an emergency department (ED) visit or inpatient admission from January 1, 2014, through December 31, 2015.^18^ Traumatic injury was identified using *International Classification of Diseases, Ninth Revision (ICD-9)* and *International Statistical Classification of Diseases and Related Health Problems, Tenth Revision (ICD-10)* codes defined based on the National Trauma Data Standard (NTDS),^19^ which included any nonsuperficial injury diagnosis, regardless of mechanism (eTable 1 in Supplement 1). The dataset included 1 year of preinjury data (January 1, 2013, to December 31, 2013) to assess underlying comorbidities and at least 1 year of postinjury data (ie, through December 31, 2016) to assess the rate of incident dementia diagnoses. Because the dataset was limited to beneficiaries continuously enrolled in Medicare Fee-for-Service during the study period, Medicare Advantage beneficiaries, who comprised 31% of all enrolled Medicare beneficiaries in 2014,^20^ were not included. Patients who were admitted under an observation status were considered inpatients if they spent at least 1 midnight in the hospital; otherwise, they were considered ED patients. We excluded patients younger than 66 years of age (n = 279 463) and patients with a preexisting diagnosis of dementia at the time of injury (n = 540 809). This study was approved by the Mass General Brigham institutional review board and granted waiver of informed consent because it posed minimal risk to participants. We followed the Strengthening the Reporting of Observational Studies in Epidemiology (STROBE) reporting guideline.^21^

### Variables

The primary outcome variable was the diagnosis of dementia, which was identified based on *ICD-9* diagnosis codes using a previously validated claims-based definition of dementia using Medicare data (eTable 2 in Supplement 1).^22^ These *ICD-9* codes were converted into *ICD-10* codes through Medicare general equivalence mapping, and both *ICD-9* and *ICD-10* codes were used to identify cases of dementia. We identified dementia diagnoses based on 1-year of preinjury data, which has reduced sensitivity compared with algorithms that use 3 years of data but has reasonable accuracy for research purposes.^23^ We determined the timing of the dementia diagnosis based on the date of the encounter at which dementia was first documented. The primary independent variable was mechanism of injury, which we dichotomized as falls vs nonfall. Falls were identified as the mechanism of injury based on previously published frameworks for identifying mechanism of injury from *ICD-9* and *ICD-10* codes.^24,25^

We assessed multiple covariates. Patient sociodemographic factors included in our analysis were age, sex (male or female), and race and ethnicity. Race and ethnicity were assessed using data reported in the Medicare Master Beneficiary Summary File, which includes race and ethnicity data reported by beneficiaries on enrollment applications. Race and ethnicity were included as covariates in analyses based on evidence that incidence of dementia varies across race and ethnic groups.^26^ Minoritized race groups were collapsed into a single category for statistical analyses due to the small number of observations. To describe underlying medical comorbidities, we calculated the Charlson Comorbidity Index using preinjury data and categorized the resulting score as 0 to 2, 3 to 5, or greater than 5.^27,28^ We described the frailty of patients using the claims-based frailty index,^29^ which ranges from 0 to 1 and was categorized as robust (<0.15), prefrail (0.15-0.24), and frail (≥0.25). However, we did not include frailty as a covariate in our multivariable model due to collinearity between frailty and the Charlson Comorbidity Index in the dataset. We assessed whether patients had an admission to a skilled nursing facility (SNF) in the year prior to injury based on Medicare claims data. We also considered hospitalization in the year prior to injury given that hospitalization may be a risk factor for incident dementia.^30,31^ To describe injury severity, we used the Injury Severity Score (ISS), which ranges from 0 to 75 with higher scores indicating more severe injuries. We categorized ISS into 4 previously described groups: mild (<9), moderate (9-15), severe (16-25), and profound (>25).^32^ We identified patients with head or neck trauma by an Abbreviated Injury Scale (AIS) score of 3 or greater for the head or neck anatomic region. We assessed whether patients had a surgical procedure for hip fractures based on procedure codes. We also assessed whether patients experienced delirium during the index encounter by examining diagnosis codes during the index encounter using previously published *ICD-9* and *ICD-10* codes.^33^

### Statistical Analysis

The primary outcome was the risk of a new dementia diagnosis, over time, after experiencing a fall. Characteristics of patients who had fallen were compared with patients with other injury mechanisms using the χ^2^ test for categorical variables and 2-tailed *t* tests for continuous variables. To evaluate our primary outcome, we performed a Cox multivariable competing risk model, using the Fine and Gray model,^34^ which compared the hazard of a dementia diagnosis for patients with a fall-related injury to those with other injury mechanisms while accounting for the competing risk of death. In the model, we controlled for patient demographics, Charlson Comorbidity Index, prior SNF admission, injury severity, presence of head or neck trauma, surgery for hip fracture, and diagnosis of delirium during the index encounter.

We performed 2 subgroup analyses. First, we assessed the hazard of new dementia diagnosis after falls among patients without a recent SNF admission, defined as not having an admission to an SNF in the year prior to the injury. Second, because our study population included ED visits with and without subsequent inpatient admission, we compared the hazard of new dementia diagnosis after falls among patients who had an ED visit only (ie, did not require inpatient hospitalization) to patients who required an inpatient admission.

Statistical significance for all statistical tests in this study was determined based on a threshold of 2-sided *P* < .05. All statistical analyses were performed using Stata version 18.0 (StataCorp) from August 2023 to July 2024.

## Results

We identified 2 453 655 patients aged 66 years and older who experienced a traumatic injury in 2014 to 2015; 1 522 656 (62.1%) were female; 124 396 (5.1%) were Black, 2 232 102 (91.0%) were White; and the mean (SD) age was 78 (8.1) years The mechanism of injury was a fall in 1 228 847 of 2 453 655 cases (50.1%); the complete distribution of mechanisms of injury is in eTable 3 in Supplement 1. Approximately one-quarter of patients (21.9% [537 513 of 2 453 655]) were admitted to a SNF in the prior year. Underlying medical comorbidities were common, with 1 231 527 patients (50.2%) having a Charlson Comorbidity Index of 1 or more. There were 326 634 patients (13.3%) with an ISS of 9 or higher. During the index clinical encounter for the injury, 119 319 patients (4.9%) underwent a surgical procedure for hip fracture, and 60 504 patients (2.5%) had a documented delirium diagnosis.

There were clinically relevant differences between patients who had fallen and patients with a different mechanism of injury (Table 1). Compared with patients with nonfall injury mechanisms, patients who fell were older (mean [SD] age: 79.5 [8.3] years vs 76.7 [7.7] years; *P* < .001), more likely to be female (67.2% vs 56.9%; *P* < .001), and more likely to be White (92.2% vs 89.8%; *P* < .001). Recent SNF admissions (28.0% vs 15.8%; *P* < .001) and recent hospitalizations (52.9% vs 40.2%; *P* < .001) were more common among patients who fell compared with patients with other mechanisms of injury. Compared with those with different injury mechanisms, patients who fell were more likely to have an ISS of at least 9 (19.0% vs 7.6%; *P* < .001). Similarly, patients who fell were more likely to have surgery for hip fracture during their index admission (8.3% vs 1.4%; *P* < .001). Although overall rates of delirium during the index clinical encounter were low, patients who fell were more likely to be diagnosed with delirium compared with those with other injury mechanisms (3.1% vs 1.8%; *P* < .001).

**Table 1.** Baseline Characteristics of Injured Older Adults in Study Cohort

Characteristics	All patients (n = 2 453 655)	Falls (n = 1 228 847)	Other injuries (n = 1 224 808)	*P* value
Age, mean (SD), y	78.1 (8.1)	79.5 (8.3)	76.7 (7.7)	<.001
Sex, No. (%)				
Male	930 999 (37.9)	402 885 (32.8)	528 114 (43.1)	<.001
Female	1 522 656 (62.1)	825 962 (67.2)	696 694 (56.9)	
Race and ethnicity, No. (%)				
Asian	23 752 (1.0)	12 696 (1.0)	11 056 (0.9)	<.001
Black	124 396 (5.1)	49 744 (4.1)	74 652 (6.1)	
Hispanic	26 155 (1.1)	12 827 (1.0)	13 328 (1.1)	
North American Native	12 696 (0.5)	5126 (0.4)	7570 (0.6)	
White	2 232 102 (91.0)	1 132 579 (92.1)	1 099 523 (89.8)	
Other^a^	34 554 (1.4)	15 875 (1.3)	18 676 (1.5)	
Frailty, No. (%)				
Robust	450 604 (18.4)	183 963 (15.0)	266 641 (21.8)	<.001
Prefrail	1 329 469 (54.2)	677 668 (55.2)	651 801 (53.2)	
Frail	673 582 (27.5)	367 216 (29.9)	306 366 (25.0)	
Charlson Comorbidity Index, No. (%)				
0	1 222 128 (49.8)	590 704 (48.1)	631 424 (51.6)	<.001
1-2	799 964 (32.6)	415 754 (33.8)	384 210 (31.4)	
≥3	431 563 (17.6)	222 389 (18.1)	209 174 (17.1)	
Recent SNF admission, No. (%)	537 513 (21.9)	343 861 (28.0)	193 652 (15.8)	<.001
Recent hospital admission, No. (%)	1 142 047 (46.5)	649 593 (52.9)	492 454 (40.2)	<.001
ISS, No. (%)				
0-8	2 127 021 (86.7)	995 349 (81.0)	1 131 672 (92.4)	<.001
9-15	259 499 (10.6)	198 286 (16.1)	61 213 (5.0)	
16-25	51 829 (2.1)	33 777 (2.8)	18 052 (1.5)	
>25	15 306 (0.6)	1435 (0.1)	13 871 (1.1)	
Head or neck trauma, No. (%)	65 791 (2.7)	47 378 (3.9)	18 413 (1.5)	<.001
Encounter type, No. (%)				
ED visit	2 022 430 (82.4)	928 150 (75.5)	1 094 280 (89.3)	<.001
Inpatient admission	431 225 (17.6)	300 697 (24.5)	130 528 (10.7)	
Surgery for hip fracture, No. (%)	119 319 (4.9)	102 302 (8.3)	17 017 (1.4)	<.001
Delirium during index encounter, No. (%)	60 504 (2.5)	38 030 (3.1)	22 474 (1.8)	<.001
Death within 1 y of fall, No. (%)	149 428 (6.1)	95 586 (7.8)	53 842 (4.4)	<.001
Dementia diagnosis within 1 y of fall, No. (%)	204 709 (8.3)	129 910 (10.6)	74 799 (6.1)	<.001

Abbreviations: ISS, injury severity score; SNF, skilled nursing facility; ED, emergency department.

^a^ Other race is a category provided by Medicare in the race variable; races included in this category are not specified.

New dementia diagnoses were more common after falls than after other injury mechanisms. In the first year after the injury, 10.6% of patients who had fallen (129 910 of 1 228 847) were diagnosed with dementia compared with 6.1% of patients with a different injury mechanism (74 799 of 1 224 808) (*P* < .001). The cumulative incidence curve of dementia diagnoses after a fall demonstrates that new diagnoses occurred gradually over the course of the year, although there was an initial sharp increase in new diagnoses in the first 1 to 2 weeks after the injury fall (Figure), which includes 21 658 dementia diagnoses across both mechanisms of injury that were made during the index encounter. The unadjusted hazard ratio [HR] of incident dementia diagnosis was 63% higher after a fall, compared with other injury mechanisms (HR, 1.63 [95% CI, 1.61-1.64]; *P* < .001). On multivariable Cox competing risk analysis, assessing the hazard of new dementia diagnosis while accounting for the competing risk of death and controlling for covariates, falls were associated with a 21% increase in the hazard of new dementia diagnosis, compared with other injury mechanisms (Table 2).

**Figure.**
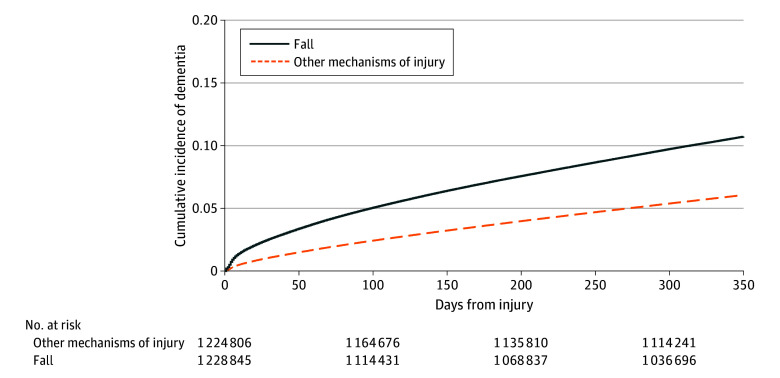
Kaplan-Meier Curves for Incident Dementia Diagnosis in First Year After a Fall Compared With Other Mechanisms of Injury

**Table 2.** Multivariable Competing Risk Model for Hazard of Dementia Diagnosis After Emergency Department Visit or Hospital Admission for Traumatic Injury Among Injured Older Adults

Variable	HR (95% CI)	
	Full cohort	Subgroup without a recent SNF admission
Mechanism of injury		
Nonfall	1 [Reference]	1 [Reference]
Fall	1.21 (1.20-1.21)	1.27 (1.26-1.28)
Age^a^	1.07 (1.07-1.07)	1.09 (1.09-1.09)
Sex		
Male	1 [Reference]	1 [Reference]
Female	0.95 (0.95-0.96)	1.05 (1.04-1.06)
Race		
White	1 [Reference]	1 [Reference]
Minoritized	1.21 (1.19-1.22)	1.15 (1.13-1.16)
Charlson Comorbidity Index		
0	1 [Reference]	1 [Reference]
1-2	0.99 (0.98-1.00)	1.02 (1.01-1.03)
≥3	0.97 (0.96-0.98)	1.02 (1.01-1.03)
SNF admission		
No recent SNF admission	1 [Reference]	NA
SNF admission within 1 y	2.00 (1.98-2.01)	NA
Hospital admission		
No recent hospital admission	1 [Reference]	1 [Reference]
Hospital admission within 1 y	1.86 (1.84-1.88)	1.88 (1.86-1.89)
ISS		
0-8	1 [Reference]	1 [Reference]
9-15	0.78 (0.77-0.79)	0.77 (0.76-0.78)
16-25	0.79 (0.76-0.81)	0.77 (0.74-0.80)
>25	1.08 (1.04-1.13)	0.94 (0.88-1.00)
Head or neck trauma		
No	1 [Reference]	1 [Reference]
Yes	1.18 (1.15-1.22)	1.35 (1.30-1.41)
Surgery for hip fracture during index encounter		
No	1 [Reference]	1 [Reference]
Yes	0.74 (0.73-0.76)	0.81 (0.79-0.83)
Delirium during index encounter		
No	1 [Reference]	1 [Reference]
Yes	1.66 (1.64-1.69)	1.60 (1.57-1.63)

Abbreviations: ISS, injury severity score; NA, not applicable; SNF, skilled nursing facility.

^a^ Age was included as a continuous variable. All other variables are categorical.

After stratifying the multivariable competing risk model by recent SNF admission, falls were associated with a 27% increased hazard of new dementia diagnosis among older adults without a recent SNF admission, compared with other mechanisms (HR, 1.27 [95% CI, 1.26-1.28]; *P* < .001). In contrast, falls were associated with a 10% increased hazard of new dementia diagnosis among patients with a recent SNF admission (HR, 1.10 [95% CI, 1.09-1.12]; *P* < .001).

Falls were a more common injury mechanism among patients with an inpatient admission compared with patients with an ED visit (69.7% [300 697 of 431 225] vs 45.9% [928 150 of 2 022 430]; *P* < .001). Furthermore, the overall rate of incident dementia diagnosis across all mechanisms of injury (including falls and nonfall mechanisms) was higher among patients with an inpatient admission compared with ED visit (12.2% [52 772 of 431 225] vs 7.5% [151 937 of 2 022 430]; *P* < .001). Unadjusted rates of incident dementia diagnosis were higher after falls compared with other injury mechanisms among both patients who required inpatient admission after the injury (13.1% [39 254 of 300 697] vs 10.4% [13 518 of 130 528]; *P* < .001) and among patients who required an ED visit alone (9.8% [90 656 of 928 150] vs 5.6%; *P* < .001). On adjusted analysis, the hazard of incident dementia diagnosis among patients who required an ED visit without admission was 29% higher after a fall compared with other injury mechanisms (HR, 1.29 [95% CI, 1.28-1.30]; *P* < .001). Among patients with inpatient admissions, the hazard of incident dementia diagnosis was 6% higher after a fall compared with other injury mechanisms (HR, 1.06 [95% CI, 1.05-1.08]; *P* < .001).

## Discussion

In this retrospective cohort study of older adult Medicare beneficiaries who experienced a traumatic injury, new dementia diagnoses were more common after falls compared with other mechanisms of injury. Falls were associated with increased risk for future dementia diagnosis even after controlling for potentially confounding factors, and this risk was greater among older adults without a recent SNF admission. Given the increased risk of a new dementia diagnosis among patients with a recent fall, our study highlights the need for cognitive screening for older adults who experience an injurious fall that results in an ED visit or hospital admission.

Other studies have also demonstrated an association between falls and incident dementia diagnosis. One study compared patients with a diagnosis of dementia to matched controls using the Swedish Twin Registry and found that the risk of falls increased sharply in the 4 years prior to dementia diagnosis and remained elevated for the first 4 years after dementia diagnosis.^13^ Another prospective cohort study found an increased risk of falls among patients with preclinical Alzheimer disease, which was identified based on the presence of biomarkers in cerebrospinal fluid.^12^ Hip fractures, which are one of the most common injuries after a fall, have also been associated with future dementia diagnosis,^14,15^ and patients with hip fractures have substantially higher rates of cerebrospinal fluid biomarkers for Alzheimer disease than other older adults.^35^ Unlike these prior studies, our study adds to the literature by assessing risk of new dementia diagnosis after falls in older adults through the use of a broad, national database.

One possible explanation for the increased risk of dementia diagnosis after a fall is that patients who fell had underlying dementia that had gone undiagnosed until the time of the fall. Undiagnosed dementia is common among older adults,^36,37^ and 11% of the new dementia diagnoses in our study occurred during the index hospitalization, suggesting that dementia was likely present but undiagnosed at the time of the fall in a subset of patients. Patients with dementia are known to be at higher risk for experiencing a fall, particularly within the first 4 years of the diagnosis.^9,10,11,13^ Experiencing a fall results in exposure to the health care system, which may lead to a formal diagnosis of dementia in patients with previously undiagnosed dementia.

Another possible explanation for the increased risk of dementia after a fall is that patients who fall and are later diagnosed with dementia have mild cognitive impairment, a precursor to dementia, at the time of the fall. Mild cognitive impairment and impairments in executive function are risk factors for falls, likely because walking requires visuospatial skills to safely navigate the environment. There is also growing evidence that motor decline—particularly among motor domains involving gait—precedes the formal diagnosis of dementia and can be seen in patients with mild cognitive impairment,^38,39,40,41^ resulting in an increased risk for falls. Therefore, patients in our study who fell may have been experiencing mild cognitive impairment at the time of the fall and subsequently progressed to developing dementia in the following year.

The association between falls and incident dementia diagnosis highlights the need for cognitive screening for older adults who experience a fall that results in an ED visit or hospital admission. These screenings are particularly important for community-dwelling older adults given that our study demonstrates that the risk of future dementia diagnosis after a fall is higher among older adults without a recent SNF admission than among those with a previous SNF admission. Cognitive screening could be performed with the Mini-Cog or AD8 instruments,^42,43^ and longer screening tools like the Montreal Cognitive Assessment (MoCA)^44^ may be appropriate for patients with high suspicion for cognitive impairment. Patients who screen positive may be referred for formal neuropsychological testing depending on the clinical context and local resources. Initial cognitive screening should be performed in the inpatient setting for patients who are admitted, unless they have a condition that would confound the screening results like delirium or traumatic brain injury (TBI). For patients who are not admitted or are unable to receive screening on the inpatient setting, outpatient cognitive screening should be performed 1 to 3 months after a fall without TBI, or 3 to 6 months after a fall with TBI. Inpatient teams will need to coordinate with the patient’s primary care physician to ensure that appropriately timed cognitive screening is performed after discharge. Specific cognitive screening guidelines for older adults after a fall should be included within the American College of Surgeons Geriatric Trauma Best Practices Guidelines. Implementing cognitive screening after injurious falls in older adults may aid in the timely diagnosis of dementia, which would allow patients and their families to plan for the future, implement supports to promote ongoing safety in the community, and gain access to treatments.^17,45^

Delirium during the index health care encounter in our study was associated with increased risk for future dementia diagnosis, which is in line with previous studies.^46^ In addition, rates of delirium were higher in patients with falls compared with other injury mechanisms. Together, these findings highlight the need to lower the risk of hospital-acquired delirium after falls. The overall rate of delirium during the index health care encounter in our study was 2.5%, which is lower than the documented incidence among hospitalized older adults.^47^ The unexpectedly low rate of delirium may be due to inaccurate coding of the diagnosis or failure of recognition.

### Limitations

Our study has limitations. This is a retrospective, observational study, which limits our ability to draw causal conclusions. In many ways, our findings suggest the association between falls and dementia may be bidirectional, with mild cognitive impairment contributing to increased risk of fall and with fall-related injury precipitating new dementia symptoms, and both circumstances resulting in new diagnosis. The claims data analyzed in this study occurred during a transition period from *ICD-9* to *ICD-10* codes, which may have resulted in modestly decreased accuracy of coding injury mechanism and higher rates of unspecified injury mechanisms.^48^ As a result, some falls may have been inaccurately coded as an unspecified accident. The sensitivity of identifying dementia diagnoses using Medicare claims is low, so it is possible that some preexisting diagnoses were missed, particularly if the patient were diagnosed more than 1 year prior to the injury and did not have Medicare claims data in the year leading up to the injury. Postinjury dementia diagnoses may also have been missed for the same reasons. The unexpectedly low rate of delirium during the index health care encounter in our study is a limitation of our study and is a known weakness of using claims data to identify delirium.^49^ In addition, we do not have access to prescription data, so we cannot control for potentially inappropriate medications that could increase both the risk of falls and delirium. The data in this study are from 2014 to 2015 due to a prolonged data acquisition process. However, we do not believe that the underlying association between falls and future dementia diagnosis would have changed over the past 10 years. A key strength of our study is the use of Medicare data, which increases generalizability because it is a broad national sample of older adults.

## Conclusions

This cohort study found that new dementia diagnoses were common after falls, with 10.6% of older adults being diagnosed with dementia in the first year after a fall. The frequency of incident dementia diagnoses after a fall highlights the need for cognitive screening for older adults who require an ED visit or hospital admission for an injurious fall. Such screening could aid in the timely diagnosis of dementia, which is a priority of the CDC Healthy Brain Initiative and would allow patients and their families to implement supports to ensure their safety in the community.^17,45^ Implementing cognitive screening after falls will require a multidisciplinary effort involving the breadth of clinicians who care for older adults after a fall, ranging from trauma surgeons to geriatricians and primary care physicians. Cognitive screening recommendations should be included in the American College of Surgeons Geriatric Trauma Best Practices Guidelines and other guidelines concerning the care of injured older adults.

## Notes

Supplement 1. **eTable 1.** National Trauma Data Standard ICD-9 and ICD-10 Codes for Identifying Traumatic Injuries **eTable 2.** ICD-9 Codes Used to Identify Dementia Diagnoses **eTable 3.** Other Mechanisms of Injury
eReferences

Supplement 2.
Data Sharing Statement

## References

[1] Moreland B, Kakara R, Henry A. Trends in nonfatal falls and fall-related injuries among adults aged ≥65 years - United States, 2012-2018. MMWR Morb Mortal Wkly Rep. 2020;69(27):875-881. doi:10.15585/mmwr.mm6927a5 32644982 PMC7732363

[2] Hsia RY, Wang E, Saynina O, Wise P, Pérez-Stable EJ, Auerbach A. Factors associated with trauma center use for elderly patients with trauma: a statewide analysis, 1999-2008. Arch Surg. 2011;146(5):585-592. doi:10.1001/archsurg.2010.311 21242421 PMC3121677

[3] Tinetti ME, Williams CS. Falls, injuries due to falls, and the risk of admission to a nursing home. N Engl J Med. 1997;337(18):1279-1284. doi:10.1056/NEJM199710303371806 9345078

[4] Gill TM, Murphy TE, Gahbauer EA, Allore HG. Association of injurious falls with disability outcomes and nursing home admissions in community-living older persons. Am J Epidemiol. 2013;178(3):418-425. doi:10.1093/aje/kws554 23548756 PMC3816345

[5] Gill TM, Murphy TE, Gahbauer EA, Allore HG. The course of disability before and after a serious fall injury. JAMA Intern Med. 2013;173(19):1780-1786. doi:10.1001/jamainternmed.2013.9063 23958741 PMC3812391

[6] Florence CS, Bergen G, Atherly A, Burns E, Stevens J, Drake C. Medical costs of fatal and nonfatal falls in older adults. J Am Geriatr Soc. 2018;66(4):693-698. doi:10.1111/jgs.15304 29512120 PMC6089380

[7] American College of Surgeons. Statement on older adult falls and falls prevention. Published September 1, 2019. Accessed October 17, 2023. https://www.facs.org/about-acs/statements/older-adult-falls-and-falls-prevention/

[8] Centers for Disease Control and Prevention. STEADI - older adult fall prevention. Published May 17, 2023. Accessed October 17, 2023. https://www.cdc.gov/steadi/index.html

[9] Montero-Odasso M, Speechley M. Falls in cognitively impaired older adults: implications for risk assessment and prevention. J Am Geriatr Soc. 2018;66(2):367-375. doi:10.1111/jgs.15219 29318592

[10] van Doorn C, Gruber-Baldini AL, Zimmerman S, ; Epidemiology of Dementia in Nursing Homes Research Group. Dementia as a risk factor for falls and fall injuries among nursing home residents. J Am Geriatr Soc. 2003;51(9):1213-1218. doi:10.1046/j.1532-5415.2003.51404.x 12919232

[11] Muir SW, Gopaul K, Montero Odasso MM. The role of cognitive impairment in fall risk among older adults: a systematic review and meta-analysis. Age Ageing. 2012;41(3):299-308. doi:10.1093/ageing/afs012 22374645

[12] Stark SL, Roe CM, Grant EA, . Preclinical Alzheimer disease and risk of falls. Neurology. 2013;81(5):437-443. doi:10.1212/WNL.0b013e31829d8599 23803314 PMC3776538

[13] Zhang L, Wang J, Dove A, Yang W, Qi X, Xu W. Injurious falls before, during and after dementia diagnosis: a population-based study. Age Ageing. 2022;51(12):afac299. doi:10.1093/ageing/afac299 36580561 PMC9799250

[14] Hsu WWQ, Zhang X, Sing CW, . Hip fracture as a predictive marker for the risk of dementia: a population-based cohort study. J Am Med Dir Assoc. 2022;23(10):1720.e1-1720.e9. doi:10.1016/j.jamda.2022.07.013 35988591

[15] Su L, Liao Y, Liu X, Xie X, Li Y. Increased risk of dementia among people with a history of fractures: a systematic review and meta-analysis of population-based studies. Front Neurol. 2023;14:1185721. doi:10.3389/fneur.2023.1185721 37545728 PMC10400716

[16] Montero-Odasso MM, Kamkar N, Pieruccini-Faria F, ; Task Force on Global Guidelines for Falls in Older Adults. Evaluation of clinical practice guidelines on fall prevention and management for older adults: a systematic review. JAMA Netw Open. 2021;4(12):e2138911. doi:10.1001/jamanetworkopen.2021.38911 34910151 PMC8674747

[17] Centers for Disease Control and Prevention. Advancing early detection - The National Healthy Brain Initiative. Published July 13, 2023. Accessed April 11, 2024. https://www.cdc.gov/aging/healthybrain/issue-maps/early-detection.html

[18] Jarman MP, Jin G, Weissman JS, . Association of trauma center designation with postdischarge survival among older adults with injuries. JAMA Netw Open. 2022;5(3):e222448. doi:10.1001/jamanetworkopen.2022.2448 35294541 PMC8928003

[19] American College of Surgeons Committee on Trauma. National Trauma Data Standard: Data Dictionary, 2015 Admissions. Accessed October 19, 2023. https://www.facs.org/media/3lelgko5/ntds-data-dictionary-2015.pdf

[20] Ochieng N, Biniek JF, Freed M, Damico A, Published TN. Medicare Advantage in 2023: enrollment update and key trends. KFF. Published August 9, 2023. Accessed April 11, 2024. https://www.kff.org/medicare/issue-brief/medicare-advantage-in-2023-enrollment-update-and-key-trends/

[21] von Elm E, Altman DG, Egger M, Pocock SJ, Gøtzsche PC, Vandenbroucke JP; STROBE Initiative. The Strengthening the Reporting of Observational Studies in Epidemiology (STROBE) statement: guidelines for reporting observational studies. Lancet. 2007;370(9596):1453-1457. doi:10.1016/S0140-6736(07)61602-X 18064739

[22] Taylor DH Jr, Østbye T, Langa KM, Weir D, Plassman BL. The accuracy of Medicare claims as an epidemiological tool: the case of dementia revisited. J Alzheimers Dis. 2009;17(4):807-815. doi:10.3233/JAD-2009-1099 19542620 PMC3697480

[23] McCarthy EP, Chang CH, Tilton N, Kabeto MU, Langa KM, Bynum JPW. Validation of claims algorithms to identify Alzheimer’s disease and related dementias. J Gerontol A Biol Sci Med Sci. 2022;77(6):1261-1271. doi:10.1093/gerona/glab373 34919686 PMC9159657

[24] Hedegaard H, Johnson RL, Garnett MF, Thomas KE. The 2020 International Classification of Diseases, 10th Revision, Clinical Modification Injury Diagnosis Framework for Categorizing Injuries by Body Region and Nature of Injury. Centers for Disease Control and Prevention. Published December 28, 2020. Accessed August 23, 2024. https://www.cdc.gov/nchs/data/nhsr/nhsr150-508.pdf 33395385

[25] McLoughlin E, Annest JL, Fingerhut LA, . Recommended framework for presenting injury mortality data. MMWR Morb Mortal Wkly Rep. 1997;46(RR-14). Accessed November 27, 2023 https://stacks.cdc.gov/view/cdc/6769/9301976

[26] Kornblith E, Bahorik A, Boscardin WJ, Xia F, Barnes DE, Yaffe K. Association of race and ethnicity with incidence of dementia among older adults. JAMA. 2022;327(15):1488-1495. doi:10.1001/jama.2022.3550 35438728 PMC9020215

[27] Charlson ME, Pompei P, Ales KL, MacKenzie CR. A new method of classifying prognostic comorbidity in longitudinal studies: development and validation. J Chronic Dis. 1987;40(5):373-383. doi:10.1016/0021-9681(87)90171-8 3558716

[28] Romano PS, Roos LL, Jollis JG. Adapting a clinical comorbidity index for use with ICD-9-CM administrative data: differing perspectives. J Clin Epidemiol. 1993;46(10):1075-1079. doi:10.1016/0895-4356(93)90103-8 8410092

[29] Kim DH, Schneeweiss S, Glynn RJ, Lipsitz LA, Rockwood K, Avorn J. Measuring frailty in Medicare data: development and validation of a claims-based frailty index. J Gerontol A Biol Sci Med Sci. 2018;73(7):980-987. doi:10.1093/gerona/glx229 29244057 PMC6001883

[30] Bohn B, Lutsey PL, Misialek JR, . Incidence of dementia following hospitalization with infection among adults in the Atherosclerosis Risk in Communities (ARIC) Study Cohort. JAMA Netw Open. 2023;6(1):e2250126. doi:10.1001/jamanetworkopen.2022.50126 36622673 PMC9857407

[31] James BD, Wilson RS, Capuano AW, . Cognitive decline after elective and nonelective hospitalizations in older adults. Neurology. 2019;92(7):e690-e699. doi:10.1212/WNL.0000000000006918 30635482 PMC6382369

[32] Bolorunduro OB, Villegas C, Oyetunji TA, . Validating the Injury Severity Score (ISS) in different populations: ISS predicts mortality better among Hispanics and females. J Surg Res. 2011;166(1):40-44. doi:10.1016/j.jss.2010.04.012 20828742

[33] Israni J, Lesser A, Kent T, Ko K. Delirium as a predictor of mortality in US Medicare beneficiaries discharged from the emergency department: a national claims-level analysis up to 12 months. BMJ Open. 2018;8(5):e021258. doi:10.1136/bmjopen-2017-021258 29730630 PMC5942463

[34] Fine JP, Gray RJ. A proportional hazards model for the subdistribution of a competing risk. J Am Stat Assoc. 1999;94(446):496-509. doi:10.1080/01621459.1999.10474144

[35] Oh ES, Blennow K, Bigelow GE, . Abnormal CSF amyloid-β42 and tau levels in hip fracture patients without dementia. PLoS One. 2018;13(9):e0204695. doi:10.1371/journal.pone.0204695 30252906 PMC6155555

[36] Amjad H, Roth DL, Sheehan OC, Lyketsos CG, Wolff JL, Samus QM. Underdiagnosis of dementia: an observational study of patterns in diagnosis and awareness in US older adults. J Gen Intern Med. 2018;33(7):1131-1138. doi:10.1007/s11606-018-4377-y 29508259 PMC6025653

[37] Anstey KJ, von Sanden C, Luszcz MA. An 8-year prospective study of the relationship between cognitive performance and falling in very old adults. J Am Geriatr Soc. 2006;54(8):1169-1176. doi:10.1111/j.1532-5415.2006.00813.x 16913981

[38] Camicioli R, Howieson D, Oken B, Sexton G, Kaye J. Motor slowing precedes cognitive impairment in the oldest old. Neurology. 1998;50(5):1496-1498. doi:10.1212/WNL.50.5.1496 9596020

[39] Kueper JK, Speechley M, Lingum NR, Montero-Odasso M. Motor function and incident dementia: a systematic review and meta-analysis. Age Ageing. 2017;46(5):729-738. doi:10.1093/ageing/afx084 28541374

[40] Skillbäck T, Blennow K, Zetterberg H, . Slowing gait speed precedes cognitive decline by several years. Alzheimers Dement. 2022;18(9):1667-1676. doi:10.1002/alz.12537 35142034 PMC9514316

[41] Verghese J, Robbins M, Holtzer R, . Gait dysfunction in mild cognitive impairment syndromes. J Am Geriatr Soc. 2008;56(7):1244-1251. doi:10.1111/j.1532-5415.2008.01758.x 18482293 PMC2574944

[42] Galvin JE, Roe CM, Powlishta KK, . The AD8: a brief informant interview to detect dementia. Neurology. 2005;65(4):559-564. doi:10.1212/01.wnl.0000172958.95282.2a 16116116

[43] Borson S, Scanlan JM, Chen P, Ganguli M. The Mini-Cog as a screen for dementia: validation in a population-based sample. J Am Geriatr Soc. 2003;51(10):1451-1454. doi:10.1046/j.1532-5415.2003.51465.x 14511167

[44] Nasreddine ZS, Phillips NA, Bédirian V, . The Montreal Cognitive Assessment, MoCA: a brief screening tool for mild cognitive impairment. J Am Geriatr Soc. 2005;53(4):695-699. doi:10.1111/j.1532-5415.2005.53221.x 15817019

[45] Robinson L, Tang E, Taylor JP. Dementia: timely diagnosis and early intervention. BMJ. 2015;350:h3029. doi:10.1136/bmj.h3029 26079686 PMC4468575

[46] Leighton SP, Herron JW, Jackson E, Sheridan M, Deligianni F, Cavanagh J. Delirium and the risk of developing dementia: a cohort study of 12 949 patients. J Neurol Neurosurg Psychiatry. 2022;93(8):822-827. doi:10.1136/jnnp-2022-328903 35606105 PMC9304115

[47] Inouye SK, Westendorp RGJ, Saczynski JS. Delirium in elderly people. Lancet. 2014;383(9920):911-922. doi:10.1016/S0140-6736(13)60688-1 23992774 PMC4120864

[48] Sebastião YV, Metzger GA, Chisolm DJ, Xiang H, Cooper JN. Impact of ICD-9-CM to ICD-10-CM coding transition on trauma hospitalization trends among young adults in 12 states. Inj Epidemiol. 2021;8(1):4. doi:10.1186/s40621-021-00298-x 33487175 PMC7830822

[49] McCoy TH Jr, Snapper L, Stern TA, Perlis RH. Underreporting of delirium in statewide claims data: implications for clinical care and predictive modeling. Psychosomatics. 2016;57(5):480-488. doi:10.1016/j.psym.2016.06.001 27480944

