# Supplementary material for: Risk of Dementia Diagnosis After Injurious Falls in Older Adults
Source: JAMA Netw Open. 2024 Sep 30;7(9):e2436606. doi: 10.1001/jamanetworkopen.2024.36606 (PMC11443352; doi:10.1001/jamanetworkopen.2024.36606)
Supplement: Supplement 1. — eTable 1. National Trauma Data Standard ICD-9 and ICD-10 Codes for Identifying Traumatic Injuries eTable 2. ICD-9 Codes Used to Identify Dementia Diagnoses eTable 3. Other Mechanisms of Injury eReferences [file jamanetwopen-e2436606-s001.pdf]

Supplemental Online Content

Ordoobadi AJ, Dhanani H, Tulebaev SR, Salim A, Cooper Z, Jarman MP. Risk of dementia diagnosis after injurious falls in older adults. *JAMA Netw Open.* 2024;7(9):e2436606. doi:10.1001/jamanetworkopen.2024.36606

eTable 1. National Trauma Data Standard ICD-9 and ICD-10 Codes for Identifying Traumatic Injuries

eTable 2. ICD-9 Codes Used to Identify Dementia Diagnoses

eTable 3. Other Mechanisms of Injury

This supplemental material has been provided by the authors to give readers additional information about their work.

© 2024 Ordoobadi AJ et al. *JAMA Network Open*.

eTable 1: National Trauma Data Standard ICD-9 and ICD-10 Codes for Identifying Traumatic Injuries*

	**Included Codes**	**Excluded**
**ICD-9**	800-959.9<br>905-909<br>910-924<br>930-939	Superficial Injury (905-909.9, 910-924.9, 930-939.9)<br>Foreign Body in Orifice<br>Late Effects of Injury
**ICD-10**	S00-S99<br>T07<br>T14<br>T20-T28<br>T30-T32<br>T79	Superficial Injury (S00, S10, S20, S30, S40, S50, S60, S70, S80, S90)<br>Foreign Body in Orifice<br>Late Effects of Injury

Abbreviations: ICD-9, International Classification of Diseases, Ninth Revision; ICD-10, International Statistical Classification of Diseases and Related Health Problems, Tenth Revision.

*Table adapted from: Jarman MP, Jin G, Weissman JS, et al. Association of Trauma Center Designation With Postdischarge Survival Among Older Adults With Injuries. JAMA Netw Open. 2022;5(3):e222448. doi:10.1001/jamanetworkopen.2022.2448

© 2024 Ordoobadi AJ et al. *JAMA Network Open*.

eTable 2: ICD-9 Codes Used to Identify Dementia Diagnoses

	**Included Codes**
**ICD-9***	331.0<br>331.1<br>331.2<br>331.7<br>290.0<br>290.1<br>290.10<br>290.11<br>290.12<br>290.13<br>290.20<br>290.21<br>290.3<br>290.40<br>290.41<br>290.42<br>290.43<br>294.0<br>294.1<br>294.8<br>797
**ICD-10**	*Consists of the above ICD-9 codes converted through general equivalence mapping to ICD-10 codes*

Abbreviations: ICD-9, International Classification of Diseases, Ninth Revision; ICD-10, International Statistical Classification of Diseases and Related Health Problems, Tenth Revision.

*Included ICD-9 codes are from: Taylor DH, Østbye T, Langa KM, Weir D, Plassman BL. The accuracy of Medicare claims as an epidemiological tool: the case of dementia revisited. J Alzheimers Dis JAD. 2009;17(4):807-815. doi:10.3233/JAD-2009-1099.

eTable 3: Other mechanisms of injury

**Mechanism***	**Events, n (%)**
Fall	1,228,847 (50)
Unspecified/other	784,286 (32)
Cut/pierce	112,167 (4.6)
Overexertion	96,351 (3.9)
Struck by/against	93,869 (3.8)
All transport	78,503 (3.2)
Natural/environmental	37,894 (1.5)
Machinery	10,785 (0.4)
Fire/burn	7,803 (0.3)
Firearm	1,476 (0.06)
Poisoning	1,234 (0.05)
Suffocation	250 (0.01)
Drowning	190 (0.01)

*Categorization of mechanism of injury is based on Centers for Disease Control and Prevention ICD Injury Code Matrices.[1,2]

References

1. Hedegaard H, Johnson RL, Garnett MF, Thomas KE. The International Classification of Diseases, 10th Revision, Clinical Modification (ICD–10–CM) external cause-of-injury framework for categorizing mechanism and intent of injury. National Health Statistics Reports; no 136. Hyattsville, MD: National Center for Health Statistics. 2019. Available from: https://www.cdc.gov/nchs/data/nhsr/nhsr136-508.pdf
2. Centers for Disease Control and Prevention. Recommended framework for presenting injury mortality data. MMWR 46(RR14):1–30. 1997. Available from: https://www.cdc.gov/mmwr/preview/mmwrhtml/00049162.htm.

© 2024 Ordoobadi AJ et al. *JAMA Network Open*.
