# Supplementary material for: Risk of Dementia Diagnosis After Injurious Falls in Older Adults
Source: JAMA Netw Open. 2024 Sep 30;7(9):e2436606. doi: 10.1001/jamanetworkopen.2024.36606 (PMC11443352; doi:10.1001/jamanetworkopen.2024.36606)
Supplement: Supplement 2. — Data Sharing Statement [file jamanetwopen-e2436606-s002.pdf]

Data Sharing Statement

Ordoobadi. Risk of Dementia Diagnosis After Injurious Falls in Older Adults. *JAMA Netw Open*. Published September 30, 2024. doi:10.1001/jamanetworkopen.2024.36606

Data
Data available: No

Additional Information
Explanation for why data not available: This study uses Medicare claims data and cannot be shared in accordance with data use agreements.
